# PRKACB is downregulated in non-small cell lung cancer and exogenous PRKACB inhibits proliferation and invasion of LTEP-A2 cells

**DOI:** 10.3892/ol.2013.1294

**Published:** 2013-04-08

**Authors:** YONG CHEN, YING GAO, YE TIAN, DA-LI TIAN

**Affiliations:** 1 Departments of Thoracic Surgery, The Fourth Affiliated Hospital of China Medical University, Shenyang, Liaoning 110032, P.R. China; 2 Pathology, The Fourth Affiliated Hospital of China Medical University, Shenyang, Liaoning 110032, P.R. China

**Keywords:** protein kinase cAMP-dependent catalytic β, non-small cell lung cancer, proliferation, apoptosis, invasion

## Abstract

Protein kinase cAMP-dependent catalytic β (PRKACB) is a member of the Ser/Thr protein kinase family and a key effector of the cAMP/PKA-induced signal transduction involved in numerous cellular process, including cell proliferation, apoptosis, gene transcription, metabolism and differentiation. In the present study, the expression pattern of PRKACB in non-small cell lung cancer (NSCLC) and the effect of PRKACB upregulation on cell proliferation, apoptosis and invasion were investigated. PRKACB mRNA and protein expression was analyzed in the NSCLC tissue and corresponding normal tissues of 30 cases, using quantitative RT-PCR and western blot analysis. A plasmid containing full-length PRKACB was transfected into LTEP-A2 cells to further investigate the effects of PRKACB overexpression on proliferation, apoptosis and invasion of the transfected cells, which were examined using 3-(4,5-dimethylthiazol-2-yl)-2,5-diphenyltetrazolium bromide (MTT), colony formation, flow cytometry and Transwell assays. The results revealed that the NSCLC tissues exhibited much lower levels of PRKACB mRNA and protein compared with their corresponding normal tissues. The upregulation of PRKACB decreased the numbers of proliferative, colony and invasive cells, while the apoptotic rates of transfected cells were increased. These data indicate that PRKACB is downregulated in NSCLC tissues and that upregulation of PRKACB may be an effective way to prevent the progression of NSCLC.

## Introduction

Lung cancer is the most commonly diagnosed type of cancer in males and the leading cause of cancer mortality in each gender in economically developed and developing countries (1). Non-small cell lung carcinoma (NSCLC) accounted for ∼85% of the all lung cancer cases (2). Standard lung cancer treatment modalities include surgery, chemotherapy, targeted therapy and radiation therapy; however, not all patients benefit from routine therapy. The overall 5-year survival rate of lung cancer patients remains relatively low at ∼15% (2). Therefore, the identification of useful biomarkers and exploration of novel therapeutic targets are necessary and demanding tasks.

The protein kinase cAMP-dependent catalytic β (PRKACB) gene is located at chromosome site 1p31.1 and encodes cAMP-dependent protein kinase A (PKA) catalytic subunit β. The PRKACB protein is a member of the Ser/Thr protein kinase family and a key effector of the cAMP/PKA-induced signal pathway that is involved in numerous cellular processes, including cell proliferation, apoptosis, gene transcription, metabolism and differentiation (3). Typically, PKA is an inactive holoenzyme consisting of two catalytic (C) subunits bound to a regulatory (R) subunit dimer. When four cAMP molecules bind the R subunits, the C subunits are released (4) and free active catalytic subunits phosphorylate serine and threonine residues on specific substrate proteins, which include C-Raf, RhoA, Src and CUTL1, that are involved in cellular proliferation, apoptosis, differentiation and invasion (5–8). In the human enzyme, four different R subunits (RIα, RIβ, RIIα and RIIβ) and four different C subunits (Cα, Cβ, Cγ and PrKX) have been identified (3). In total, ten different splice variants encoded by the PRKACB gene have been found and a certain number of these were revealed to be expressed in human brain, lymphoid and neuronal tissues (9–11). Multiple PRKACB subunits have also been observed in human prostate specimens and it appears that the PRKACB variants play varying roles in proliferation and differentiation of prostate cancer progression (12). It has been demonstrated that transcription of PRKACB may be directly activated by c-MYC, which is associated with tumorigenesis by the promotion of cell proliferation (13). It has also been shown that a variant of PRKACB phosphorylates the p75 neutrophin receptor (p75NTR) and regulates its localization to lipid rafts (14). PRKACB was identified as a candidate gene that is directly or indirectly involved in apoptosis in human mantle cell lymphoma (MCL) tumors (15). In addition, a novel interaction between PRKACB, the cell cycle and apoptosis regulatory protein-1 (CARP-1) was identified and confirmed by glutathione-S-transferase (GST) pull-down experiments in brain tissue (16). However, limited information is known with regard to its expression and role in human NSCLC.

The present study aimed to assess the role of PRKACB in the development and progress of human NSCLC. The mRNA and protein expression patterns of PRKACB were first examined in the NSCLC and corresponding normal tissues. Moreover, plasmid vectors containing full-length PRKACB and transfected human adenocarcinoma LTEP-A2 cells were constructed to increase the PRKACB expression. The effects of PRKACB upregulation on cell proliferation, clonogenicity, apoptosis and invasion were then investigated in the LTEP-A2 cells.

## Materials and methods

### Tissue samples and patients

NSCLC tissues (12 cases of lung squamous cell carcinoma tissues, 18 cases of lung adenocarcinoma tissues; 22 of these 30 cases presented with lymph node metastasis) and their corresponding normal tissues (30 cases) were collected from 30 patients who underwent surgery at the Department of Thoracic Surgery, The Fourth Affiliated Hospital of China Medical University, Shenyang, Liaoning, China, between 2008 and 2012. All tumor tissues were diagnosed histopathologically by at least two trained pathologists. Written informed consent was obtained from all patients prior to surgery and the study protocol was approved by the Institutional Review Board for the use of Human Subjects at China Medical University (Shenyang, China). None of the patients received pre-operative chemotherapy or radiation therapy. Surgically-removed tumors and matched normal tissues were immediately frozen in liquid nitrogen and kept at −80°C until the extraction of the RNA and protein.

### RNA extraction and real-time RT-PCR

Total RNA from the frozen tissues was isolated using TRIzol reagent (Takara Bio Inc., Dalian, Liaoning, China). Quantitative real-time polymerase chain reaction (QPCR) was conducted using SYBR Premix Ex Taq (Takara Bio Inc.) in a total volume of 20 *μ*l using a 7300 Real-Time PCR System (Applied Biosystems, Foster City, CA, USA). The PCR conditions were; denaturation at 95°C for 30 sec, followed by a further 40 cycles of denaturation at 95°C for 5 sec, and finally annealing at 60°C for 31 sec. The sequences of the primer pairs are as follows: PRKACB forward, 5′-AGTGGTTTGCCACGACAGATTG-3′; and reverse, 5′-TTGCTGGTACCAGAGCCTCTAA-3′; GAPDH forward 5′-GCACCGTCAAGGCTGAGAAC-3′; and reverse, 5′-TGGTGAAGACGCCAGTGGA-3′. GAPDH was used as the reference gene. The relative levels of gene expression were calculated using the 2^−ΔCt^ method (ΔCt = Ct of PRKACB − Ct of GAPDH) and the fold change of gene expression was calculated by the 2^−ΔΔCt^ method. All experiments were repeated in triplicate.

### Western blot analysis

The total protein from the frozen tissues was extracted in a lysis buffer (Beyotime Biotechnology, Haimen, Jiangsu, China) and the protein content was determined using the bicinchoninic acid (BCA) assay (Beyotime Biotechnology). A total of 80 *μ*g total protein was separated by sodium dodecyl sulfate polyacrylamide gel electrophoresis (SDS-PAGE) and then transferred onto polyvinylidene fluoride (PVDF) membranes. Subsequent to blocking with 5% bovine serum albumin (BSA), PRKACB antibody (1:500; Santa Cruz) and GAPDH antibody (1:500; Santa Cruz) were incubated on membranes for PRKACB and GAPDH protein overnight at 4°C. The membranes were then incubated for 2 h at 37°C with goat anti-rabbit IgG (1:4000; Beijing Biosynthesis Biotechnology Co., Ltd., Beijing, China). Immunoreactive strips were identified using the enhanced chemiluminescence (ECL) system (Beyotime Biotechnology) following the manufacturer’s instructions. The DNR Imaging System (DNR Bio-Imaging Systems, Israel) was used to identify the specific bands, and the optical density of each band was measured using Image J software (NIH, Bethesda, MD, USA). The ratio between the integrated optical density (IOD) of PRKACB and GAPDH of the same sample was calculated as the relative content and expressed graphically.

### Cell culture and transfection

Lung adenocarcinoma LTEP-A2 cells were obtained from the Shanghai Cell Bank (Shanghai, China). The cells were grown in RPMI-1640, supplemented with 10% fetal bovine serum (FBS; Hyclone, USA) and placed in an incubator with 5% CO_2_ at 37°C. To increase the PRKACB expression for subsequent experiments, the LTEP-A2 cells (60–70% confluence) were transfected with a plasmid containing full-length PRKACB (pEGFP-C1-PRKACB) and the vector control (pEGFP-C1; Takara Bio Inc.) for 48 h using Lipofectamine LTX with PLUS reagent (Invitrogen, Carlsbad, CA, USA), according to the manufacturer’s instructions. The experiments were repeated at least three times. The efficiency of the transfection in the experiments was >50%. Following 36–48 h of transfection, the cells with high PRKACB expression were confirmed by real-time RT-PCR and western blot analysis.

### 3-(4,5-dimethylthiazol-2-yl)-2,5-diphenyltetrazolium bromide (MTT) assay

The MTT assay was used to evaluate the proliferation of the transfected cells. The cells were detached and seeded into five 96-well plates (5×10^3^ cells/100*μ*l/well) in parallel and transfected with PRKACB and the vector control. During the following 4 days, the absorbance of one indicated plate was examined each day, and the cells in the other plates were cultured continuously. A total of 20 *μ*l MTT (5 mg/ml) was added to each well of the indicated plate, and 4 h later the liquids were removed and 150 *μ*l dimethyl sulphoxide (DMSO) was added. Following 10 min of agitation, the absorbance was measured using a microplate reader (TECAN, Männedorf, Switzerland) at 492 nm. The results were plotted as the mean ± SD of five determinations.

### Colony formation assay

The cells were transfected with PRKACB and the vector for 24 h. Thereafter, 200 cells were planted into 6-cm cell culture dishes and incubated for 14 days. The plates were stained with Giemsa, and colonies with >50 cells were counted.

### Cell apoptosis assay

Cell apoptosis was examined by flow cytometry using an Annexin V-PE/7-aminoactinomycin D (7-AAD) apoptosis detection kit (KeyGEN Biotech., Nanjing, China), following the manufacturer’s instructions. At 24 h post-transfection, the cells were washed twice in ice-cold PBS. The cells (100 *μ*l; 1×10^5^) were gently mixed with 50 *μ*l binding buffer and 5 *μ*l 7-AAD and then incubated for 15 min at room temperature in the dark. Subsequent to supplementation with another 450 *μ*l binding buffer, 1 *μ*l Annexin V-PE was added to the cells, which were then incubated for another 15 min at room temperature in the dark. Cell apoptosis was detected using a flow cytometer. The results are representative of three individual experiments.

### Cell invasion assay

The cell invasion assay was performed using a 24-well Transwell chamber (Costar, Cambridge, MA, USA). At 24 h post-transfection, the cells (4×10^4^) were seeded in the upper chamber of a 8-*μ*m pore size insert pre-coated with Matrigel (BD Biosciences-Pharmingen, San Diego, CA, USA), and cultured in RPMI-1640 without FBS for a further 24 h. The cells were allowed to migrate towards the medium containing 10% FBS in the bottom chamber. The non-migratory cells on the upper membrane surface were removed with a cotton tip, and the migratory cells attached to the lower membrane surface were fixed with 4% paraformaldehyde and stained with crystal violet (Sigma, St. Louis, MO, USA). The number of invaded cells were counted in 10 randomly selected power fields under a microscope (magnification, ×200) (Olympus CK30; Olympus, Tokyo, Japan). The experiments were performed in triplicate.

### Statistical analysis

The SPSS for Windows version 17.0 statistical analysis software (SPSS, Inc., Chicago, IL, USA) was applied to complete the data processing. A paired-samples t-test was used to compare the differences between the PRKACB expression in the NSCLC and corresponding normal tissues. One-way ANOVA was used to compare the differences in PRKACB expression in the transfected LTEP-A2 cells or controls. All data are represented as the mean ± SD. P<0.05 was considered to indicate a statistically significant difference.

## Results

### Expression of PRKACB mRNA and protein in human NSCLC tissues and their corresponding normal tissues

**Figure 1:**
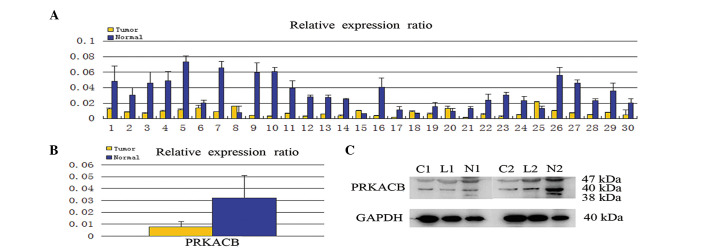
Expression of PRKACB in NSCLC specimens. (A) Real-time PCR analyses of PRKACB mRNA in NSCLC tissues and their corresponding normal tissues. (B) The average PRKACB expression for all studied tumor samples and their corresponding normal tissues. (C) PRKACB protein expression by western blot analysis. C1 and C2, NSCLC tissues; L1 and L2, metastasis lymph nodes; N1 and N2, corresponding normal tissues. PRKACB, protein kinase cAMP-dependent catalytic β; NSCLC, non-small cell lung cancer.

The PRKACB mRNA expression was first quantitatively determined in the clinical samples using real-time RT-PCR. Of the 30 patients, 25 (83.3%) demonstrated a lower expression level of PRKACB mRNA in the NSCLC tissues compared with the corresponding normal tissues (Fig. 1A). In addition, the mean expression value of the PRKACB mRNA in NSCLC tissues (relative ratio of PRKABC/GAPDH; 0.007677±0.004608) was significantly weaker than the value in the normal tissues (0.031936±0.018996; P<0.05; Fig. 1B). Consistent with the mRNA level, the protein levels of PRKACB were downregulated in the NSCLC tissues compared with the normal tissues (0.350±0.124 vs. 0.964±0.245, respectively; P<0.05; Fig. 1C). The study also demonstrated that PRKACB protein expression was downregulated in lymph node metastasis tissues (data not shown).

### PRKACB upregulation inhibits proliferation and clonogenicity in NSCLC cells

**Figure 2:**
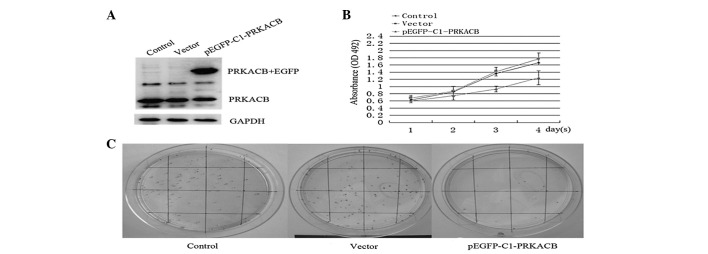
Inhibition of cell proliferation by PRKACB transfection. (A) PRKACB transfected TEP-A2 cells exhibited increased PRKACB expression in comparison to those vector-transfected and control cells by western blot assays. (B) Growth curves showed the lower tendency to proliferation of PRKACB transfected cells compared with vector and control groups by MTT assay of the 4 days examined. (C) Assessment of clonogenic potentials of the PRKACB transfected LTEP-A2 cells. The number of colonies formed by PRKACB-transfected cells was far less than that of vector-transfected and control cells. Data are represented as the mean ± SD of three independent experiments. PRKACB, protein kinase cAMP-dependent catalytic β; MTT, 3-(4,5-dimethylthiazol-2-yl)-2,5-diphenyltetrazolium bromide.

To elucidate the biological role of PRKACB during carcinogenesis, the physiological effects of PRKACB upregulation on cell proliferation and clonogenicity were examined using the LTEP-A2 cells. Fig. 2A shows the overexpression of PRKACB in the transfected cells. The study showed that 3 days after PRKACB transfection, the absorbance values in the PRKACB, vector and control groups were 0.93±0.08, 1.41±0.12 and 1.36±0.09, respectively (one-way ANOVA, P<0.05). The growth curve shows that the cells transfected with pEGFP-C1-PRKACB grew more slowly than the empty vector-transfected cells and control group cells, indicating that PRKACB inhibits proliferation in NSCLC cells (Fig. 2B).

The colony formation efficiencies of the LTEP-A2 cells transfected with PRKACB and the vector control for 24 h were compared next. In total, 200 cells were planted on 6-cm cell culture dishes. At two weeks post-transfection, the plates were stained with Giemsa and colonies with >50 cells were counted. The numbers of cell colonies in the PRKACB, vector and control groups were 23.42±5.38, 89.28±7.15 and 86.85±6.86, respectively (one-way ANOVA, P<0.05; Fig. 2C). These results showed that the increased expression of PRKACB significantly inhibited the colony formation efficiencies of the LTEP-A2 cells. Collectively, these data suggest that PRKACB may act as a negative regulator of cell growth and that its downregulation plays a significant role in NSCLC carcinogenesis.

### Elevated apoptotic rate in PRKACB transfected cells

**Figure 3:**
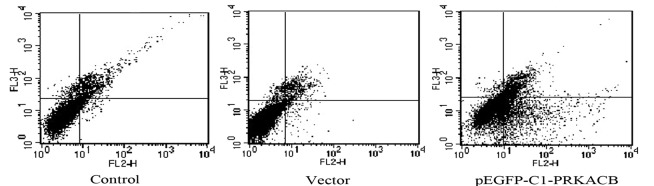
PRKACB-promoted cell apoptosis. The apoptotic rate of PRKACB upregulated cells was increased in contrast to the vector-transfected and control cells. The results are indicated as the mean ± SD of three individual tests. PRKACB, protein kinase cAMP-dependent catalytic β.

PRKACB has been considered to prevent the overgrowth of cells by inducing cell apoptosis (15,16). Therefore, apoptosis was examined following PRKACB transfection using Annexin V-PE/7-AAD assay and flow cytometry. It was confirmed that PRKACB was upregulated in the transfected cells. The apoptotic rates of the LTEP-A2 cells in the PRKACB, vector and control groups were 24.43±3.42, 4.39±1.63 and 3.48±1.44%, respectively (one-way ANOVA, P<0.05; Fig. 3). The results showed that apoptosis was significantly induced in the PRKACB overexpressed cells.

### Effect of PRKACB upregulation on the invasive potential of transfected cells

**Figure 4:**
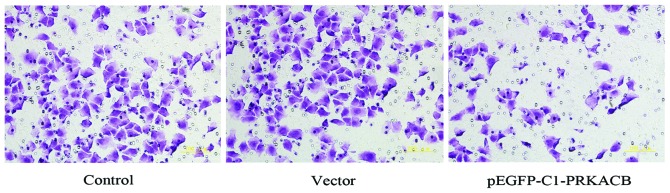
Interruption of cell invasion by PRKACB transfection. The number of invasive cells in the upregulated PRKACB group was significantly reduced compared with the vector and control groups. The values are the mean ± SD of three replicates. PRKACB, protein kinase cAMP-dependent catalytic β.

It has been acknowledged that PKA may inhibit RhoA signaling, which has been implicated in the process of tumor cell invasion and metastasis (6). To determine whether PRKACB expression further affects the invasion of LTEP-A2, the present study compared the invasive ability of the three cell groups. The number of invasive cells in the PRKACB, vector and control groups were 83.6±9.5, 156.9±13.7 and 154.2±12.9, respectively (one-way ANOVA, P<0.05; Fig. 4). These results show that the increased expression of PRKACB significantly inhibited the invasion of the LTEP-A2 cells, as demonstrated by the Matrigel invasion assay.

## Discussion

The PRKACB gene is located at the 1p31.1 chromosome site and encodes PKA catalytic subunit β, which is a member of the Ser/Thr protein kinase family. As a key effector of the cAMP/PKA-induced signaling pathway, the free C subunits phosphorylate serine and threonine residues on specific substrate proteins and regulate a wide range of cellular processes. Previous studies have identified the loss of 1p31.1 in MCL patients and the MCL cell line. PRKACB has been identified as an apoptotic candidate gene and it appears that decreased expression of PRKACB is implicated in human MCL (15). PRKACB tissue-specific expression has also been found in human brain, neuronal, lymphoid and prostate cancer tissues, and has been reported to be correlated with cellular proliferative or differentiation processes (9–12). However, there are no studies investigating the role of PRKACB in lung cancer. In the present study, the mRNA and protein levels of PRKACB were downregulated in the human NSCLC tissues compared with their corresponding normal tissues. These results suggest that PRKACB has a critical effect in the tumorigenesis and aggression of NSCLC.

A recent study discovered a novel interaction between PRKACB, the cell cycle and CARP-1; this was confirmed by GST pull-down experiments in brain tissue (16). A study has also demonstrated that PRKACB interacts with p75NTR, which phosphorylates p75NTR at Ser304 (14). In the majority of cases, the most prominent biological function of p75NTR is that it induces cell death and induces the activity of the JNK-p53-Bax apoptosis pathway and other proteins that regulate cell death, such as NRIF (17). PKA-mediated phosphorylation at Ser304 has been shown to promote the translocation of p75NTR to lipid rafts and to regulate the downstream signals of p75NTR, including the inactivation of RhoA, which has been implicated in the process of tumor cell invasion and metastasis. In addition, PKA may also directly inhibit RhoA signaling; when Ser188 is phosphorylated, RhoA becomes inactive and thereby induces characteristic morphological changes, causing cell rounding (6). These data suggest that decreased PRKACB is associated with cellular apoptosis, invasion and metastasis. With the aim of assessing the role of PRKACB in the development and progress of human NSCLC, the present study examined the effects of exogenously-transfected PRKACB on the apoptosis and invasion of LTEP-A2 cells. Consistent with the aforementioned findings, the present study concluded that the upregulation of PRKACB increased the number of apoptotic cells and decreased the number of invasive cells. The results demonstrate the potential role of PRKACB in the development and progression of human NSCLC.

As previously described, PKA was able to induce the signal pathway that is involved in numerous cellular process, including cell proliferation, apoptosis and gene transcription (3). cAMP-mediated PKA activation has been shown to have anti-proliferative effects in a number of cell types, including thyroid papillary carcinoma, ovarian epithelial cancer, breast cancer and malignant glioma cells (18–26). These anti-proliferative effects are mainly associated with the negative regulation of the Ras-Raf-MEK-ERK signaling pathway by interfering with the activation of Raf-1 directly or via Ras in the Raf-1 pathway (5,24,27). Several other mechanisms have been proposed to explain the anti-proliferative effects of activated PKA on various other cells and tissues, including a decrease in the expression level of cyclin D3 and an upregulation of the amount of p27kip1 (26). PKA is able to inhibit CUTL1-mediated proliferation and migration (8), as well as the LPA stimulation of SRF by promoting the dissolution of F-actin (19). In this study, we further examined the effects of exogenously transfected PRKACB on the proliferation of LTEP-A2 cells. The observation that the upregulation of PRKACB induces decreased proliferation of the LTEP-A2 cells is consistent with a negative role for PKA in the proliferation of these cells. Exogenously expressed PRKACB may effectively inhibit the progression of lung cancer. However, the fact that the excess of free PRKACB subunits may generate signals different from those generated by the cAMP/PKA-induced signal pathway cannot be excluded. It has also been previously shown that the activation of PKA has either proliferative or anti-apoptotic effects in cultured cells, and that these opposite responses may be due to the existence of cell type-specific targets of this signaling pathway (12,13).

The present study demonstrated that PRKACB was down-regulated in human NSCLC tissues. Decreased PRKACB appears to be associated with cellular apoptosis, invasion and proliferation. However, the molecular mechanisms for these processes remain primarily unknown. Increased PRKACB expression is possibly an effective inhibitor of lung cancer. The upregulation of PRKACB may provide a useful strategy for future NSCLC inhibitory therapies.
