# [Expression of Concern] PRKACB is downregulated in non-small cell lung cancer and exogenous PRKACB inhibits proliferation and invasion of LTEP-A2 cells

**DOI:** 10.3892/ol.2026.15573

**Published:** 2026-04-07

**Authors:** Yong Chen, Ying Gao, Ye Tian, Da-Li Tian

Oncol Lett 5: 1803–1808, 2013; DOI: 10.3892/ol.2013.1294

Following the publication of the above paper, it was drawn to the Editor's attention by a concerned reader that, regarding the cell invasion assay data shown in Fig. 4 on p. 1806, the Control and Vector data panels contained an overlapping section, such that data which were intended to show the results from differently performed experiments had apparently been derived from the same original source.

The authors have been contacted by the Editorial Office to offer an explanation for this apparent anomaly in the presentation of the data in this paper. Owing to the fact that the Editorial Office has been made aware of potential issues surrounding the scientific integrity of this paper, we are issuing an Expression of Concern to notify readers of this potential problem while the Editorial Office continues to investigate this matter further.

